# U.S. Filipino Adults Have Elevated Prevalence of Hypertension Across the Adult Lifespan: Findings From a Cross-Sectional Electronic Health Record Study

**DOI:** 10.1016/j.focus.2024.100211

**Published:** 2024-02-23

**Authors:** Nancy P. Gordon, Irvin C. Lien, Jamal S. Rana, Joan C. Lo

**Affiliations:** 1 Division of Research, Kaiser Permanente Northern California, Oakland, California; 2 The Permanente Medical Group, Oakland, California; 3 Department of Medicine, Kaiser Permanente Oakland Medical Center, Oakland, California; 4 Department of Cardiology, Kaiser Permanente East Bay, Oakland, California; 5 Department of Health System Science, Kaiser Permanente Bernard J. Tyson School of Medicine, Pasadena, California

**Keywords:** Hypertension prevalence, racial health disparities, ethnic health disparities, Filipino adults, Chinese adults, South Asian adults

## Abstract

**Introduction:**

The prevalence of hypertension increases with age and differs by race and ethnicity. Among U.S. Asian adults, prevalence is higher for Filipino adults than for other major Asian subgroups, but whether this disparity exists across the adult lifespan is unknown. This study examined hypertension prevalence by age decade, comparing Filipino adults with South Asian, Chinese, Black, Hispanic, and White adults.

**Methods:**

This cross-sectional study used 2015–2016 electronic health record data from a Northern California integrated healthcare delivery system for 1,839,603 adults aged 30–79 years, including 128,124 Filipino adults. Hypertension was defined by diagnosis codes. Sex-specific prevalence was calculated by race and ethnicity overall and by 10-year age decade from ages 30–39 years to 70–79 years. The prevalence of hypertension among 5 racial and ethnic groups was compared within each decade (with Filipino as the reference), adjusting for age, English language, diabetes, smoking, and weight category.

**Results:**

Decade-specific prevalence of hypertension among Filipino men and women, respectively, was 9.7% and 8.5% for ages 30–39 years, 26.0% and 23.9% for ages 40–49 years, 45.9% and 44.4% for ages 50–59 years, 65.4% and 63.9% for ages 60–69 years, and 82.1% and 82.9% for ages 70–79 years. Across all age decades, hypertension prevalence among Filipino adults largely tracked with Black adults and was much higher than among South Asian, Chinese, White, and Hispanic adults. This pattern remained after adjusting for covariates, with the largest differences observed for adults aged <60 years.

**Conclusions:**

Similar to Black adults, Filipino adults have persistently higher hypertension prevalence than South Asian, Chinese, Hispanic, and White adults across the adult lifespan. These findings underscore the importance of surveillance and prevention efforts for this high-risk Asian group beginning in early adulthood.

## HIGHLIGHTS

• Hypertension prevalence in Filipino adults is high, in the range of that for Black adults.
• Hypertension prevalence is higher in Filipino than in White and Hispanic adults.
• Hypertension prevalence is higher in Filipino than in South Asian and Chinese adults.
• These differences in prevalence are observed across age decades from ages 30 to 79 years.

## INTRODUCTION

Hypertension is an important risk factor for cardiovascular disease and cardiovascular mortality.^1^ Numerous studies document racial and ethnic differences in hypertension prevalence,^2^, ^3^, ^4^, ^5^ including increased risk and earlier age of onset among Black and Hispanic adults than among non-Hispanic White (White) adults.^6^ However, fewer studies have examined hypertension prevalence among U.S. Asian adults. Earlier findings from the 2003–2005 National Health Interview Survey showed that hypertension prevalence among Filipino adults approached that of White adults, with both groups having prevalence higher than that among Chinese and Asian Indian adults; accounting for age, sociodemographic factors, and health status, Filipino adults had 1.2-fold higher odds of hypertension than White adults.^7^ Survey data from the Behavioral Risk Factor Surveillance System (2013/2015/2017) showed an overall lower prevalence of hypertension among Asian Americans than among White adults but significant variation among Asian subgroups (higher for Filipino and Japanese adults).^8^ In addition, a study of northern California adults with primary care visits in 2010–2012 demonstrated higher hypertension prevalence among Filipino and Black adults than among other Asian subgroups, White, and Hispanic adults.^9^

Consistent with these findings by other investigators, the authors of this study previously observed that the (age-standardized) prevalence of hypertension was significantly higher for Filipino adult members of a Northern California health plan than for Chinese, South Asian, White, and Hispanic adults, with a prevalence similar to that of Black adults.^10^ In this study, the authors used the same population cohort to further investigate whether the same racial and ethnic trends are evident within age decade strata from age 30 years to age 79 years. They also examined whether observed differences in hypertension prevalence were independent of other measured factors associated with hypertension risk, including obesity, smoking, and diabetes.^11^^,^^12^

## METHODS

### Study Population

This cross-sectional study used 2015–2016 electronic health record (EHR) data from a community-dwelling clinical cohort of adults who in 2016 were members of Kaiser Permanente Northern California (KPNC),^10^ an integrated healthcare delivery system with a sociodemographically diverse membership of >3.4 million adults. The cohort of 1,839,603 members aged 30–79 years was comprised of 1,011,592 non-Hispanic White (47.4% male); 145,772 Black (42.8% male); 347,071 Hispanic (48.8% male); 128,124 Filipino (42.3% male); 126,321 Chinese (45.3% male); and 80,723 South Asian (51.7% male) adults, including large numbers within each 10-year age strata among the 3 largest U.S. Asian subgroups (Table 1).

**Table 1:** Number of Men and Women by Race and Ethnicity Within Each Age Decade

Age decade, years	White	Black	Hispanic	Filipino	Chinese	South Asian
Men						
30–39	85,644 (17.9%)	11,606 (18.6%)	46,987 (27.7%)	10,909 (20.1%)	12,586 (22.0%)	15,977 (38.3%)
40–49	90,724 (18.9%)	14,001 (22.5%)	48,758 (28.8%)	13,492 (24.9%)	12,784 (22.3%)	12,122 (29.1%)
50–59	117,752 (24.6%)	17,094 (27.4%)	40,032 (23.6%)	13,350 (24.6%)	13,526 (23.6%)	7,288 (17.5%)
60–69	115,175 (24.0%)	13,083 (21.0%)	22,783 (13.5%)	11,011 (20.3%)	12,608 (22.0%)	4,290 (10.3%)
70–79	70,296 (14.7%)	6,523 (10.5%)	10,883 (6.4%)	5,460 (10.1%)	5,716 (10.0%)	2,032 (4.9%)
Total number	479,591	62,317	169,443	54,222	57,220	41,709
Women						
30–39	92,890 (17.5%)	16,800 (20.1%)	49,268 (27.7%)	15,819 (21.4%)	15,560 (22.5%)	16,738 (42.9%)
40–49	95,511 (18.0%)	18,599 (22.3%)	48,422 (27.3%)	18,362 (24.9%)	16,343 (23.7%)	10,579 (27.1%)
50–59	126,165 (23.7%)	21,365 (25.6%)	40,633 (22.9%)	17,532 (23.7%)	16,279 (23.6%)	6,211 (15.9%)
60–69	133,336 (25.1%)	17,335 (20.8%)	25,811 (14.5%)	14,563 (19.7%)	14,524 (21.0%)	3,826 (9.8%)
70–79	84,099 (15.8%)	9,356 (11.2%)	13,494 (7.6%)	7,626 (10.3%)	6,395 (9.3%)	1,660 (4.3%)
Total number	532,001	83,455	177,628	73,902	69,101	39,014

### Measures

Information on race and ethnicity was derived from EHR and KPNC survey data sources (or language preference data if race and ethnicity data were not available). Surname-based assignment was conducted in a small subset, as previously described.^10^ Details on assignment to respective racial and ethnic groups by age decade are provided in Appendix Table A1 (available online).

Hypertension prevalence was determined by problem list diagnosis codes and by outpatient encounter diagnosis codes in 2015–2016, as previously described^10^ (Appendix Table A2, available online).

### Statistical Analysis

The authors first examined sex-specific hypertension prevalence by race and ethnicity for ages 30–79 years, age standardized to the 2016 U.S. population, and then compared unadjusted sex-specific hypertension prevalence within age decades. Modified log-Poisson regression was used to calculate adjusted prevalence ratios within each age decade, comparing hypertension prevalence in White, Black, Hispanic, Chinese, and South Asian adults with that in Filipino adults, after controlling for age, English language preference (as a proxy for acculturation), diabetes,^10^ smoking status, and ethnic weight category (underweight, healthy, overweight, obesity Classes 1–3) on the basis of standard BMI thresholds for White, Black, and Hispanic adults (<18.5, 18.5 to <25, 25 to <30, 30 to <35, 35 to <40, ≥40 kg/m^2^) and lower BMI thresholds for Asian adults (<18.5, 18.5 to <23, 23 to <27.5, 27.5 to <32.5, 32.5 to <37.5, ≥37.5 kg/m^2^).^13^^,^^14^ These covariates were included owing to their known association with hypertension and availability in the EHR. A category for missing data was created for smoking status (3.3%) and BMI (38.0%). Analyses were conducted in 2022–2023 using SAS, Version 9.4 (SAS Institute, Cary, NC).

### Ethics

This study was approved by the KPNC IRB with a waiver of informed consent.

## RESULTS

The age-standardized prevalence of hypertension among men and women aged 30–79 years, respectively, was 40.9% and 39.6% for Filipino, 41.4% and 43.5% for Black, 31.1% and 27.1% for South Asian, 29.9% and 28.3% for Hispanic, 28.2% and 23.5% for White, and 23.8% and 20.7% for Chinese adults. Table 2 further shows the demographic and clinical characteristics of the cohort examined in this study, including findings by race and ethnicity.

**Table 2:** Study Cohort Characteristics Among Men and Women, by Race and Ethnicity

Characteristic	White	Black	Hispanic	Filipino	Chinese	South Asian
Men	*n*=479,591	*n*=62,317	*n*=169,443	*n*=54,222	*n*=57,220	*n*=41,709
Mean age±SD, years	54.3±13.2	52.7±12.6	48.5±12.1	51.9±12.7	52.0±13.1	45.6±11.9
Hypertension^a^	32.0%	43.0%	24.6%	41.3%	24.1%	21.0%
Diabetes	12.1%	19.9%	16.4%	25.3%	12.3%	16.4%
Smoking status^b^						
Current smoker	10.7%	15.0%	10.3%	12.9%	9.0%	6.5%
Nonsmoker	89.3%	85.0%	89.7%	87.1%	91.0%	93.5%
BMI category^c^^,^^d^						
Underweight	0.3%	0.5%	0.1%	0.3% [0.3%]	1.1% [1.1%]	0.5% [0.5%]
Healthy weight	20.0%	15.7%	11.3%	24.3% [8.8%]	47.2% [22.6%]	34.2% [13.9%]
Overweight	42.4%	37.1%	41.6%	49.4% [42.6%]	40.8% [51.3%]	48.0% [49.2%]
Obesity Level 1	24.7%	28.1%	31.0%	19.0% [34.8%]	8.9% [20.3%]	14.0% [28.7%]
Obesity Level 2	9.1%	12.5%	11.6%	5.2% [9.5%]	1.5% [3.6%]	2.8% [6.0%]
Obesity Level 3	3.6%	6.1%	4.5%	1.7% [4.0%]	0.4% [1.1%]	0.5% [1.7%]
Preferred language						
English	98.2%	99.1%	68.4%	96.2%	77.0%	94.8%
Other	0.7%	0.1%	30.7%	2.7%	20.0%	2.8%
Unknown	1.1%	0.8%	0.9%	1.0%	3.0%	2.5%
Women	*n*=532,001	*n*=83,455	*n*=177,628	*n*=73,902	*n*=69,101	*n*=39,014
Mean age±SD, years	54.9±13.4	52.5±12.9	49.0±12.6	51.7±12.8	51.5±12.9	44.7±11.9
Hypertension^a^	27.8%	44.9%	23.9%	39.5%	20.1%	15.9%
Diabetes	8.7%	17.2%	14.1%	20.1%	8.6%	10.8%
Smoking status						
Current smoker	7.5%	10.4%	4.1%	4.4%	1.6%	0.9%
Nonsmoker	92.5%	89.6%	95.9%	95.6%	98.4%	99.1%
BMI category^b^						
Underweight	1.5%	0.7%	0.5%	1.1% [1.1%]	4.5% [4.5%]	1.4% [1.4%]
Healthy weight	34.5%	15.8%	20.9%	40.2% [21.4%]	64.9% [44.7%]	40.9% [22.5%]
Overweight	30.4%	28.7%	34.2%	37.7% [41.0%]	23.4% [36.0%]	37.7% [40.7%]
Obesity Level 1	18.6%	26.0%	25.1%	15.0% [24.9%]	5.4% [11.4%]	15.0% [25.2%]
Obesity Level 2	9.4%	16.4%	12.4%	4.6% [7.9%]	1.3% [2.4%]	3.9% [7.4%]
Obesity Level 3	5.7%	12.4%	7.0%	1.5% [3.5%]	0.4% [0.9%]	1.2% [2.9%]
Preferred language						
English	98.7%	99.6%	73.1%	97.3%	73.7%	92.6%
Other	0.9%	0.2%	26.6%	2.3%	25.3%	6.4%
Unknown	0.4%	0.2%	0.3%	0.4%	1.0%	1.0%

a Percentage with hypertension unadjusted for age (age-adjusted hypertension prevalence is detailed in the Results section).

b Smoking data were missing in 4.5% and 2.3% of White, 4.5% and 2.3% of Black, 6.2% and 2.1% of Hispanic, 4.7% and 2.0% of Filipino, 10.3% and 3.9% of Chinese, and 6.8% and 2.7% of South Asian men and women, respectively.

c Standard BMI thresholds for all groups and additional Asian-specific BMI thresholds for Asian groups are in [brackets].

d BMI data were missing in 39.9% and 36.2% of White, 40.9% and 37.6% of Black, 43.2% and 34.3% of Hispanic, 39.6% and 34.1% of Filipino, 44.6% and 37.3% of Chinese, and 40.0% and 35.2% of South Asian men and women, respectively.

**Figure 1:**
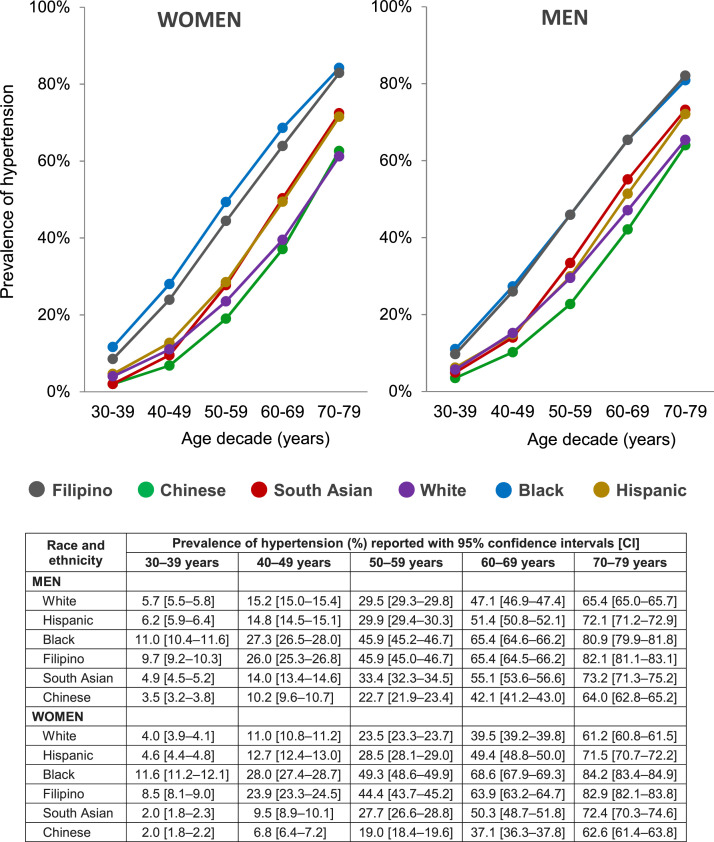
Prevalence of hypertension by sex, age decade, and race and ethnicity.

The prevalence of hypertension within each age decade increased from ages 30–39 years to ages 70–79 years across all racial/ethnic groups for both men and women (Figure 1). Within each age decade, the prevalence of hypertension among Filipino adults was similar (men) or modestly lower (women) than among Black adults, with prevalence among both groups exceeding those of the other racial and ethnic groups by at least 10 percentage points across most of the age decades examined.

**Figure 2:**
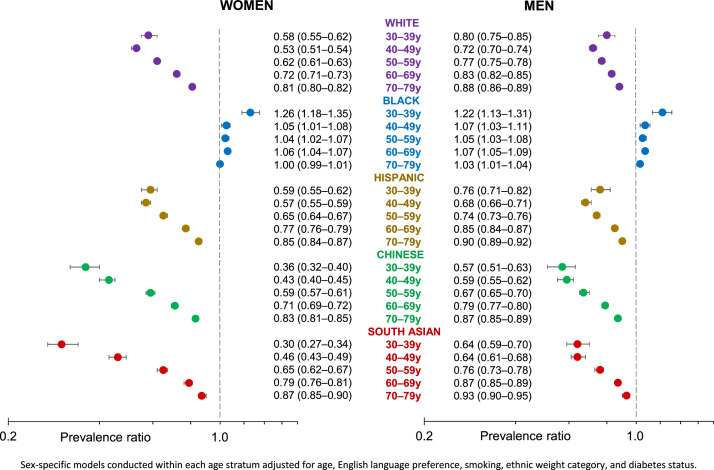
Prevalence ratio for hypertension by sex and age decade comparing White, Black, Hispanic, Chinese, and South Asian adults with Filipino adults.

Figure 2 shows the prevalence ratios from multivariable analyses conducted within each age decade, adjusted for age, ethnic-specific BMI category, diabetes, smoking, and English language preference. The adjusted hypertension prevalence among Filipino adults was higher than in Chinese, South Asian, Hispanic, and White adults within each age decade, with wider disparities observed across the first 3 decades (30–59 years), particularly among women. Black adults had a higher adjusted prevalence than Filipino adults, but this was only slightly higher (3%–7%) for the last 4 decades (40–79 years).

## DISCUSSION

In this and an earlier report based on the same source cohort,^10^ the age-standardized prevalence of hypertension among Filipino men and women was substantially higher than among Chinese, South Asian, Hispanic, and White adults and approached the prevalence among Black adults. These results are consistent with findings from another independent primary care cohort also based in Northern California with a much smaller number of Filipino adults (*n*=6,120).^9^ In this study, comprising >128,000 Filipino and 126,000 Chinese adults and nearly 81,000 South Asian adults, the authors extend their prior findings^10^ and document a much higher prevalence of hypertension among Filipino than among Chinese, South Asian, White, and Hispanic adults in each 10-year age stratum across the adult lifespan. Differences were largest under ages 50–60 years and independent of age, weight category, smoking, and diabetes status. In contrast, minor or no significant differences in adjusted prevalence were seen between Filipino and Black adults aged 40–79 years, by age decade. This study is among the first to report age-specific trends in hypertension risk among Filipino adults in relation to other major U.S. racial and ethnic groups and major U.S. Asian subgroups.

Researchers hypothesize that multiple factors may contribute to greater hypertension risk among Filipino adults, including higher rates of smoking, diet and physical inactivity, overweight or obesity, chronic conditions such as diabetes, and social factors, including stress.^15^, ^16^, ^17^, ^18^ In an earlier survey-based study of KPNC adults, the authors found that middle-aged Filipino adults not only had higher obesity and smoking rates than Chinese and White adults but also were more likely to have dietary and sleep risks associated with development of hypertension.^19^ In a survey study of 1,028 Filipino immigrants in the New York City area (average age of 51.9 years, 31.8% male, 53% with hypertension), older age, male sex, longer residence in the U.S., BMI ≥23.0 kg/m^2^, elevated blood glucose, family history of hypertension, and self-reported fair or poor health status were predictors of hypertension.^17^ In the present study, the higher prevalence of hypertension among Filipino adults was independent of age, smoking, diabetes, and weight status (when measured), but data on lifestyle, family, and social factors were not available. Collectively, the findings of this study and those of others support hypertension screening and prevention efforts for Filipino adults beginning in early adulthood, combined with culturally tailored education and lifestyle counseling.^16^^,^^20^

### Limitations and Strengths

This study has some limitations, including reliance on clinical diagnoses, which may result in underestimation of hypertension prevalence. There may be possible race and ethnicity misclassification.^10^ The authors also do not have data pertaining to social and behavioral determinants of health, including education, income, dietary sodium intake, and physical activity, as well as more recently identified factors such as religion or spirituality.^21^^,^^22^ Data for BMI were also incomplete, and information on waist circumference was not available. Finally, these findings may not be generalizable to uninsured and very low-income populations or populations in other U.S. regions. Nonetheless, this study had several strengths, including large numbers among Asian subgroups enabling direct comparisons within 5 age decades; use of EHR diagnosis data rather than self-report or single-day clinical measurements to assign hypertension status; inclusion of non-English speakers who are often under-represented in survey and recruited study cohorts; and a large, sociodemographically diverse population cohort receiving care from the same regional healthcare system with equal access to guideline-driven hypertension screening in primary care.

## CONCLUSIONS

Similar to Black adults, Filipino adults have persistently higher hypertension prevalence than South Asian, Chinese, Hispanic, and White adults across the adult lifespan. These findings underscore the importance of surveillance and prevention efforts for this high-risk Asian group beginning in early adulthood, similar to ongoing efforts to reach high-risk Black adults.^23^

## CRediT authorship contribution statement

**Nancy P. Gordon:** Conceptualization, Methodology, Formal analysis, Writing – original draft, Writing – review & editing, Supervision. **Irvin C. Lien:** Conceptualization, Writing – original draft, Writing – review & editing. **Jamal S. Rana:** Writing – review & editing. **Joan C. Lo:** Conceptualization, Methodology, Formal analysis, Writing – original draft, Writing – review & editing.

## Appendix Supplementary materials

Image, application 1

## Footnotes

Supplementary material associated with this article can be found in the online version at doi:10.1016/j.focus.2024.100211.
