# Supplementary material for: U.S. Filipino Adults Have Elevated Prevalence of Hypertension Across the Adult Lifespan: Findings From a Cross-Sectional Electronic Health Record Study
Source: AJPM Focus. 2024 Feb 23;3(3):100211. doi: 10.1016/j.focus.2024.100211 (PMC11021886; doi:10.1016/j.focus.2024.100211)
Supplement: Supplementary file 1 [file mmc1.pdf]

Appendix:

Table A1. Percentages of adults in the study cohort whose racial and ethnic group was assigned based on race, ethnicity, or language preference data from electronic health records or survey questionnaires[1]

	30-39 years	40-49 years	50-59 years	60-69 years	70-79 years
White	100.0%	100.0%	100.0%	100.0%	100.0%
Black	100.0%	100.0%	100.0%	100.0%	100.0%
Hispanic	100.0%	100.0%	100.0%	100.0%	100.0%
Filipino	74.8%	78.4%	83.1%	89.5%	94.8%
Chinese	62.9%	72.5%	82.0%	90.4%	95.6%
South Asian	54.1%	49.4%	57.9%	69.2%	80.0%

[1] Remaining percentages for Filipino, Chinese, and South Asian adults were assigned based on vetted surname lists.

Table A2. ICD codes used to assign cohort to hypertension status

ICD-10 codes	ICD-9 codes
I13.0, I13.1, I13.2, I13.9, I15.0, I15.8, I15.9, I10, I11.0, I11.9, I12.0, I12.9, H35.039	362.11, 401.0, 401.1, 401.9, 402.00, 402.01, 402.10, 402.11, 402.90,402.91, 403.00, 403.01, 403.10, 403.11, 403.90, 403.91, 404.01, 404.03, 404.11, 404.13, 404.91, 404.93, 405.01, 405.09, 405.11, 405.19, 405.91, 405.99
